# Mosquito Inhibition

**Published:** 1907-06

**Authors:** W. C. Abbott

**Affiliations:** Chicago, Ill.


For Texas Medical Journal.

### Mosquito Inhibition.

BY W. C. ABBOTT, M. D., CHICAGO, ILL.

Last winter when yellow fever was frightening visitors away from the sunny Southland, a new method of preventing this disease and malaria was suggested. Believing there was merit in the idea, I took pains to make it public in the States most directly interested; but, unfortunately, the public had been "worked" scientifically by the arsenic prophylactic swindle, and the profession was not in the humor for experimenting. Nevertheless, the few reports received on the method indicated that it was at least successful enough to warrant further and more extended trial.

Briefly, the idea was that when a person had taken enough calx sulphurata (U. S. Pharmacopeia) to so saturate the body that the odor of sulphurated hydrogen emanated from the skin and breath, no mosquito or other insect could be induced to attack that person so long as such saturation endured. The proposition originated in Alaska, where persons are accustomed to defend themselves against the ferocious mosquitoes of that region by applying a solution of calx sulphurata to the exposed parts of the skin. This may be done by dissolving (suspending) 18 grains of full strength calx sulphurata in one ounce of glycerine and two ounces of water, and applying liberally to the exposed skin just before emerging from the protection of the mosquito bar. While effective, this plan is insufficient, in that the insect finds unprotected spots for entry, and the solution may be rubbed off. When the body is saturated with the sulphide there are no unprotected spots.

Saturation may be attained by taking from one-half to one grain of pure sulphide every hour while awake, the odor appearing on the breath or the skin within a very few hours. Observations covering thousands of cases have shown that this is absolutely harmless to man or infant. The writer has given two grains every two hours to children with diphtheria many times with benefit and impunity. The gas when inhaled has the power of reducing hemoglobin and even to a perilous degree; but no instance of such action has yet been reported when the salt is taken internally.

It is best to omit the medicine during the period of acid digestion, as hydrochloric acid decomposes it, setting free sulphureted hydrogen, whose odor in eructations is unpleasant to the patient. Small and frequent doses are preferable to larger ones, since too large doses may cause nausea and interfere with the persistent taking necessary to secure saturation.

The reports received on this matter to date indicate the correctness of the claim that no insect of any kind—mosquito, chigger, fly, flea, redbug, pediculus or bedbug—will attack a person so saturated. Whatever disease may be transmissible by such carriers is therefore prevented.

No harm results from such saturation, continued for weeks, beyond the possibly undesirable effect of inhibiting the sexual function which has occasionally been reported. This disappears as the saturation passes off, and no permanent effect remains. Since sexual indulgence has been recognized as predisposing to attacks of yellow fever, this suspension, while one is exposed to the malady, can not be considered objectionable.

Calx sulphurata is official in the U. S. Pharmacopeia, where it is described on page 86. This requires the presence of at least 60 per cent of calcium sulphide, with calcium sulphate and carbon in varying proportions. It is a difficult drug to handle pharmacally, and an examination of many brands found in the city pharmacies showed that but one complied with the requirements of the Pharmacopeia. Some had not even a trace of true salt, and had apparently been prepared by treating plain tablets with sulphydric acid until a faint odor had been absorbed. The recommendations herein made apply only to the true U. S. P. preparation.

There is opportunity to test this preventive widely in the regions where malaria prevails in the fall. If sulphide saturation wards off the stegomyia, it will equally inhibit the anopheles. The trial is easy, harmless and inexpensive; if successful, it renders the physician, who stalks abroad at all hours during the pestilence, immune against both diseases.

